# A High-Precision Screen-Printed Glucose Sensor with In Situ Impedance-Based HCT Correction and Temperature Compensation

**DOI:** 10.3390/bios16040193

**Published:** 2026-03-28

**Authors:** Mingxin Lu, Jie Cheng, Qinyao Lei, Jinhong Guo, Kuo Chen

**Affiliations:** 1 School of Automation and Intelligent Sensing, Shanghai Jiao Tong University, Shanghai 200240, China; 2 School of Computer Science and Technology, Chongqing University of Posts and Telecommunications, Chongqing 404100, China; chenkuo@cqupt.edu.cn

**Keywords:** electrochemical glucose sensor, hematocrit correction, in situ impedance measurement, temperature compensation, POCT

## Abstract

Hematocrit (HCT) fluctuations and ambient temperature variations are two critical interference factors limiting the accuracy of electrochemical glucose test strips in self-monitoring of blood glucose (SMBG). In this study, a high-precision screen-printed glucose sensor incorporating in situ impedance-based HCT correction and temperature compensation was developed. The system employs a time-division multiplexing strategy, integrating a normalized thermodynamic model and an in situ impedance-based HCT correction algorithm, to achieve synergistic decoupling and precise compensation of temperature and HCT interferences. Experimental results demonstrate that after multi-parameter synergistic correction, the system exhibits excellent stability across a wide temperature range (10–35 °C) and a broad HCT range (10–70%). The accuracy indicators significantly surpass ISO 15197:2013 standards. In contrast, uncorrected measurements showed deviations ranging from approximately −80% to +30% due to HCT fluctuations. This multiple correction strategy effectively resolves systematic errors in whole blood testing without increasing electrode complexity or requiring pretreatment steps, providing a robust technical solution for high-precision, low-cost personal glucose monitoring.

## 1. Introduction

Diabetes has emerged as a severe global public health challenge, and strict glycemic control is the core strategy for delaying the progression of complications [1,2]. As the cornerstone of diabetes management, Self-Monitoring of Blood Glucose (SMBG) requires detection devices capable of providing rapid and reliable diagnostic data in non-laboratory environments [3,4]. In recent years, electrochemical and photoelectrochemical sensing platforms have achieved cutting-edge advancements in the precise point-of-care detection of various physiological biomarkers [5] and glucose. Specifically, electrochemical glucose biosensors based on enzymatic catalytic reactions have distinguished themselves among various technologies due to their fast response, minimal sample volume requirements, and low manufacturing costs [6]. However, despite substantial progress in existing technologies, the measurement accuracy of electrochemical glucose test strips in practical clinical applications remains significantly limited by interferences from endogenous substances and environmental fluctuations, leading to a persistent gap between “laboratory accuracy” and “home monitoring accuracy” [7,8,9].

Among the various factors affecting the accuracy of electrochemical blood glucose monitoring, fluctuations in hematocrit (HCT) and variations in ambient temperature are widely recognized as the most dominant sources of interference. HCT not only alters the effective mass transfer rate at the electrode surface through physical obstruction and volume exclusion effects [10,11,12,13,14,15], but also directly impacts the viscosity and diffusion coefficient of the sample. Without proper correction, the blood glucose values of low-HCT samples (e.g., from anemic patients) are frequently overestimated, whereas those of high-HCT samples (e.g., from neonates) tend to be underestimated [16,17].

Currently, to meet the stringent accuracy standards of ISO 15197:2013 [18], mainstream commercial blood glucose monitoring systems commonly employ multi-frequency AC impedance spectroscopy or multi-electrode technologies to estimate HCT and perform algorithmic compensation [10]. Although these standard protocols perform well in the general population (HCT 30–55%), they still exhibit limitations when confronted with extreme physiological conditions. Multiple independent clinical evaluation reports have indicated that when samples fall within the ranges of anemia (HCT < 25%) or polycythemia (HCT > 60%, e.g., in neonates), numerous certified commercial glucometers still manifest significant measurement biases exceeding ±15% [19,20,21,22]. Such deviations are primarily attributed to the fact that commercial test strips often lack the capability to quantify the red blood cell obstruction effect in situ and in real time. Consequently, blood glucose readings remain largely unreliable in complex clinical scenarios, such as intensive care or neonatal care [23,24].

It is worth noting that existing temperature compensation strategies also encounter significant challenges. In recent years, to mitigate measurement errors induced by thermodynamic fluctuations, researchers have widely integrated in situ temperature compensation strategies into various cutting-edge biosensing platforms [25,26]. However, for complex whole blood electrochemical systems, existing single-parameter temperature compensation strategies still face immense challenges. The HCT effect and temperature fluctuations do not exist in isolation within electrochemical systems; instead, they exhibit complex coupling characteristics. From the perspective of mass transfer mechanisms, variations in ambient temperature significantly alter the viscosity of blood samples and the kinetics of enzymatic reactions [27]. At non-standard temperatures, the attenuation of the current signal is the consequence of the synergistic effects of thermodynamic factors (temperature) and geometric factors (HCT). For commercial blood glucose systems, systematic errors introduced by algorithmic corrections based on erroneous temperature parameters can reach up to 30% [22]. Therefore, in the absence of a unified thermodynamic baseline, traditional single-parameter calibration models often struggle to precisely decouple the independent effect of HCT, ultimately leading to measurement failures under extreme environments.

To overcome the aforementioned HCT and temperature effects, researchers have proposed various compensation strategies. Early physical separation techniques (e.g., red blood cell lysis or membrane filtration), while effective in eliminating erythrocyte interference, significantly increase the complexity and manufacturing cost of the test strips [28,29,30]. In recent years, although integrated approaches based on microfluidic impedance or radio frequency (RF) detection have enhanced measurement accuracy [31,32,33], these methods often rely on complex signal processing circuitry or expensive noble metal electrodes, making them difficult to implement on a large scale for low-cost, disposable test strips. Therefore, to address the limitations of existing commercial products under extreme HCT and dynamic temperature conditions, it is of significant clinical translational value to develop an integrated solution that preserves the low-cost advantages of screen-printed carbon electrodes (SPCEs) while enabling in situ calibration for both HCT and temperature.

**Figure 1:**
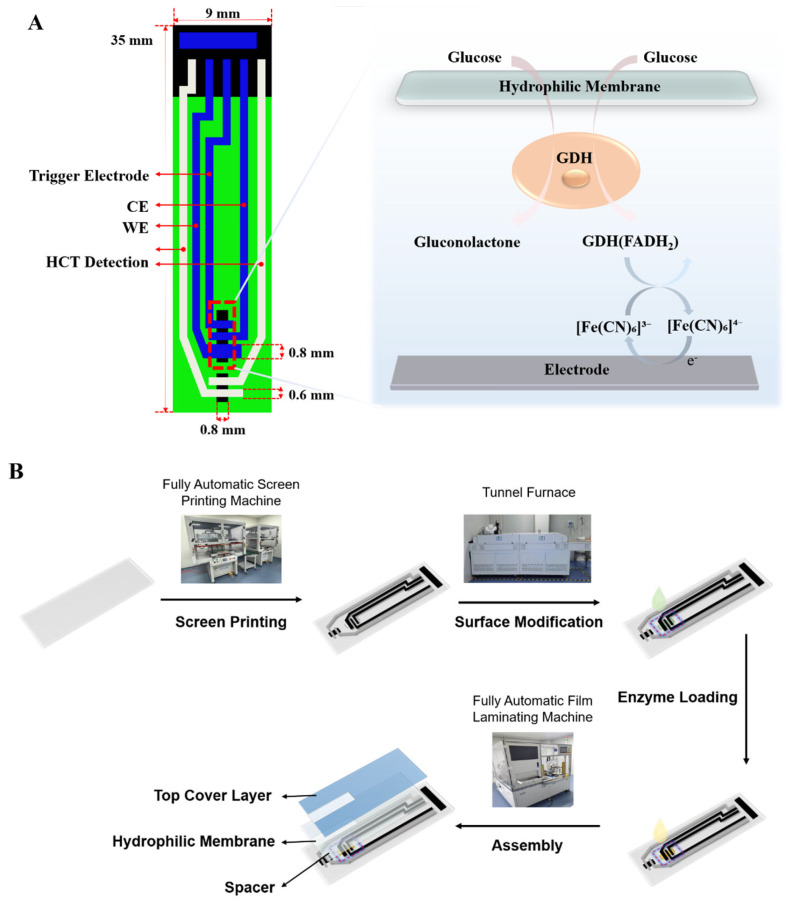
(**A**) Schematic illustration of the electrochemical whole blood glucose sensing strip with HCT detection and multiple correction strategies, and the principle of blood glucose detection. (**B**) Fabrication process and structural composition of the glucose sensing strip (arrows indicate the fabrication workflow, and droplet colors are for illustrative purposes only).

In this study, we propose a high-precision glucose sensing strip structure integrated with in situ HCT correction and temperature compensation (Figure 1A). This strip is constructed using mature screen-printing technology based on a glucose dehydrogenase (GDH)/ferricyanide reaction system. Unlike traditional hardware compensation schemes, we employed a time-division multiplexing strategy to perform AC impedance measurement (for HCT estimation) and DC chronoamperometry (for glucose detection) sequentially on the same set of electrodes, eliminating the need for additional electrode structures. On this basis, we established a temperature correction model and an impedance normalization-based HCT compensation model, successfully decoupling the composite effects of environmental temperature and HCT on the enzymatic reaction and electrochemical mass transfer processes. Performance evaluation indicates that this multiple correction strategy significantly reduces measurement deviations caused by HCT fluctuations, demonstrating excellent accuracy and stability across a wide HCT range (10–70%) and temperature range (10–35 °C), thereby providing strong technical support for the realization of low-cost, high-precision next-generation personal glucose monitoring systems.

## 2. Materials and Methods

### 2.1. Reagents and Materials

Glucose dehydrogenase (Sigma-Aldrich, St. Louis, MO, USA) and potassium ferricyanide (Xiya Reagent, Linyi, China) served as the core reaction system of the enzyme electrode, supplemented with buffer salts for the preparation of the enzyme solution. Reagents required for electrode pretreatment and the formulation of the composite sensing layer, including chitosan, cellulose, trehalose, bovine serum albumin (BSA), polyvinyl pyrrolidone (PVP), and Tween 20, were purchased from Sigma-Aldrich. Interfering substances used for testing, including bilirubin, ascorbic acid, cholesterol, triglycerides, uric acid, acetaminophen and ibuprofen were purchased from Shanghai Source Biotechnology Co., Ltd (Shanghai, China). All chemicals were of analytical grade and used without further purification. Ultrapure water was used throughout the experiments.

Venous whole blood samples were collected from healthy volunteers and treated with anticoagulants. To obtain samples with varying HCT levels, plasma and red blood cells were separated via centrifugation. Subsequently, the red blood cells were resuspended in plasma at specific ratios to prepare a series of whole blood samples covering an HCT range of 10% to 70%. Prior to testing, all blood samples were thoroughly mixed on a roller mixer and equilibrated at room temperature for at least 30 min to eliminate temperature discrepancies. Glucose control solutions spanning low, medium, and high concentration ranges were utilized for the baseline performance verification of the sensors.

### 2.2. Preparation of Glucose Sensing Strip

Disposable electrochemical glucose test strips were constructed using screen-printing technology. The strip substrate consisted of polyethylene terephthalate (PET). The electrode system included a carbon working electrode and a carbon counter electrode for glucose detection, as well as carbon electrodes for HCT detection and a carbon trigger electrode.

The fabrication process, as illustrated in Figure 1B, comprised the following steps:

Electrode Pretreatment and Enzyme Immobilization: The working electrode was first pretreated with 0.5 wt% chitosan to enhance surface hydrophilicity. The final dispensing reagent was prepared by homogeneously mixing a cellulose solution with an enzyme stock solution at a 1:1 volume ratio. The enzyme stock, prepared in PBS, precisely contained 2 KU/mL of GDH, 200 mM of potassium ferricyanide (electron mediator), bioprotectants (3% trehalose and 5 mg/mL BSA) for thermal stability, a film-forming agent (1% PVP), and a surfactant (0.05% Tween 20). Finally, 1.2 μL of this composite mixture was dispensed onto the modified electrode and dried at 50 °C for 20 min to form a robust sensing layer.

Microchannel Assembly: A composite laminate consisting of a blood-guiding spacer and a hydrophilic membrane was applied to the electrode plate to construct the sample inlet channel. Subsequently, a protective cover was laminated onto the top layer.

Cutting and Quality Control: The electrode sheets were cut into individual strips. After passing surface integrity checks and electrical continuity checks, the qualified strips were sealed and stored under dry conditions.

### 2.3. Electrochemical Measurement System and Procedure

All electrochemical measurements in this study were performed using a custom-built, miniaturized electronic device. To achieve dual-parameter detection of HCT and glucose concentration, a time-division multiplexing measurement system was established. The system interfaced with the test strips via a custom electrochemical connector, and the measurement protocol consisted of two consecutive stages:

AC Impedance Measurement: An AC sinusoidal signal with a frequency of 10 kHz and an amplitude of 40 mV (rms) was applied to measure the impedance modulus of the whole blood sample for HCT quantification.

Chronoamperometry Measurement: Immediately following the impedance measurement, the system switched to DC mode. A constant working potential of 0.5 V was applied, and the steady-state oxidation current was recorded as the glucose detection signal. To avoid the interference of the initial non-steady-state current, the current value at the 5th second was recorded as the response signal for glucose concentration.

### 2.4. Performance Evaluation and Experimental Design

HCT Effect Evaluation: Whole blood samples with varying glucose concentrations (2.6 mM–29.18 mM) were prepared, and their HCT levels were adjusted to 10%, 20%, 30%, 40%, 50%, 60%, and 70%. Sensor responses were recorded at a standard temperature (25 °C) to evaluate the extent of HCT interference on the measured current.

Temperature Effect and Compensation Experiment: The ambient temperature was set to 10 °C, 15 °C, 20 °C, 25 °C, 30 °C, and 35 °C, respectively. At each temperature point, tests were conducted after the test strips and samples reached thermal equilibrium. Current data were collected to construct the temperature compensation model.

Interference Resistance Experiment: Common blood interfering substances, including bilirubin, ascorbic acid, cholesterol, triglycerides, and uric acid, were selected. These substances were spiked at concentrations higher than physiological levels into blood samples at both hypoglycemic (4.7 mM) and hyperglycemic (12.7 mM) levels. Measurement biases were calculated to evaluate the sensor’s selectivity.

Accelerated Aging Experiment: Sealed test strips were stored continuously in a constant temperature chamber at 50 °C for 21 days. At specific time intervals, strips were retrieved to test control solutions at low (2.6 mM), medium (10.59 mM), and high (29.18 mM) concentration levels. Long-term storage stability was verified by comparing the results with those of the room-temperature storage group.

## 3. Results

### 3.1. Sensing Principle and Dual-Mode Detection Mechanism

The electrochemical sensing system constructed in this study is based on the electron transfer mechanism involving GDH and potassium ferricyanide. As illustrated in Equations (1)–(3), GDH immobilized on the working electrode specifically catalyzes the oxidation of glucose to gluconolactone, accompanied by the reduction of the enzyme cofactor FAD to $FADH_{2}$ (Equation (1)). Subsequently, the reduced enzyme transfers electrons to the oxidized mediator, converting it into the reduced mediator and regenerating the enzyme (Equation (2)). Finally, driven by the applied working potential, the reduced mediator undergoes electrochemical oxidation at the carbon electrode, generating a response current positively correlated with the substrate glucose concentration (Equation (3)).

To achieve in situ detection of HCT levels without disrupting blood cells, this system employs a time-division multiplexing measurement strategy. Prior to the biochemical reaction detection, a low-amplitude, high-frequency AC signal (10 kHz, 40 mV rms) is applied to perform impedance measurement. At this frequency, the RBC membrane exhibits significant capacitive reactance, rendering the measured impedance highly sensitive to the volume fraction of RBCs (i.e., HCT) while remaining minimally influenced by the double-layer capacitance [10,34]. Upon completion of the impedance measurement, the system immediately switches to the DC amperometric mode for the electrochemical quantification of glucose concentration. This in situ dual-mode detection design lays the foundation for subsequent signal decoupling and compensation.

(1) $$Glucose+GDH(FAD)\to Gluconolactone+GDH(FADH_{2})$$

(2) $${GDH(FADH}_{2})+{2Fe\left(CN\right)}_{6}^{3-}\to GDH\left(FAD\right)+2{Fe\left(CN\right)}_{6}^{4-}+2H^{+}$$

(3) $$2{Fe\left(CN\right)}_{6}^{4-}\overset{Electrode}{\to }{2Fe\left(CN\right)}_{6}^{3-}+{2e}^{-}$$

### 3.2. Impact of HCT on Electrochemical Response

**Figure 2:**
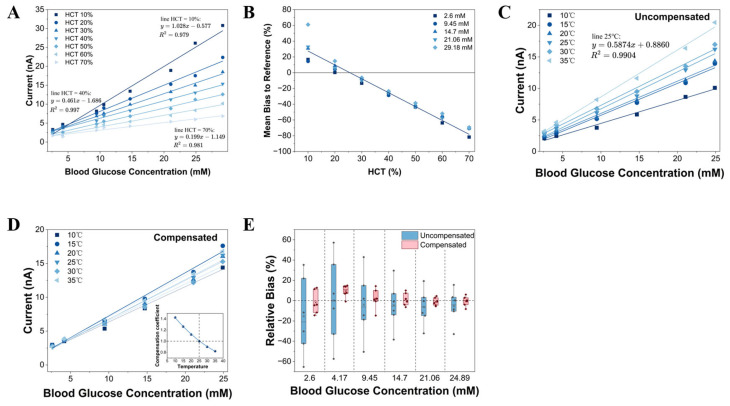
(**A**) Glucose concentration–current response curves at different HCT levels (10–70%) without calibration. (**B**) Mean bias of measured glucose values versus reference values (tested concentration range: 2.6 mM–29.18 mM, each group conducts 5 independent tests.). (**C**) Current-concentration response curves at different temperatures without temperature compensation exhibit deviations, with 25 °C selected as the baseline due to its good fit. (**D**) After temperature compensation, the current-concentration response curves at different temperatures highly converge to the 25 °C baseline, effectively suppressing systematic thermal deviations. Inset: Fitting of the normalized coefficient used in the temperature compensation model (i.e., the expanded form of Equation (4), where $k_{temp}(25\;{}^{\circ}C)=1)$. (**E**) Relative bias of measured glucose concentrations before and after temperature compensation. The scatter box plots illustrate the bias distribution across 6 distinct ambient temperatures (10–35 °C). The ’×’ symbol indicates the mean, and the horizontal line inside the box represents the median. Each data point superimposed on the boxes represents the mean value of 10 independent replicate measurements at a specific temperature condition. (concentrations were calculated using a base calibration curve established at 25 °C and 40% HCT).

HCT modulates the mass transfer kinetics of reaction substrates through volume exclusion effects and diffusion hindrance, serving as a critical variable that directly induces deviations in the response of electrochemical glucose sensors [35]. Figure 2A illustrates the current–glucose concentration response curves under varying HCT levels (10–70%). The results indicate that HCT exerts a significant modulating effect on sensor sensitivity. Specifically, the response current exhibits a marked non-linear decay trend as HCT increases. Quantitative analysis (Figure 2B) demonstrates that without correction, the HCT effect results in substantial measurement errors. Using 40% HCT as the baseline, glucose measurements in low-HCT samples (10%) were overestimated by an average of 30.13%, whereas those in high-HCT samples (70%) were underestimated by up to 76.92%. This high dependency on HCT (regression analysis R^2^ > 0.99) confirms that standalone chronoamperometry cannot satisfy the clinical accuracy requirements for whole blood analysis, necessitating the implementation of a dynamic compensation method based on HCT identification.

### 3.3. Temperature Effect and Thermodynamic Compensation

Ambient temperature fluctuations significantly interfere with the electrochemical response through a dual coupling mechanism involving kinetics and mass transfer processes. Mechanistically, an increase in temperature exponentially enhances the catalytic rate constant ($k_{cat}$) of GDH in accordance with the Arrhenius equation. Simultaneously, consistent with the Stokes–Einstein relation, it accelerates the diffusive mass transfer of substrates and electron mediators by reducing solution viscosity. Experiments indicate that within the clinical temperature range of 10 °C to 35 °C, the sensor response current exhibits significant positive thermal sensitivity (Figure 2C). This thermal effect introduces a systematic bias independent of glucose concentration, which must be decoupled at the front-end of signal processing to achieve standardization.

Distinct from adding additional, dedicated temperature-sensing microelectrodes or integrating functional thermosensitive layers on the disposable test strip itself, this study established a normalized compensation strategy using 25 °C as the reference state. Although enzyme catalysis intrinsically follows exponential kinetic laws, regression analysis based on empirical data within the target temperature range confirms that a second-order polynomial model provides extremely high goodness of fit (R^2^ > 0.9992) (Figure 2D Inset) while ensuring computational efficiency. Moreover, the temperature coefficient $k_{temp}$ exhibits independence from glucose concentration (*p* > 0.05). Consequently, this system employs a normalized temperature coefficient ($k_{temp}$) to map the measured current $I_{T}$ at any temperature T to the standard equivalent current $I_{ref}\;(I_{ref}=I_{T}*k_{temp})$. Based on the normalization condition centered at 25 °C (i.e., $k_{temp}(25\;{}^{\circ}C)=1$), the compensation model is established as shown in Equation (4):

(4) $$k_{temp}=0.0004{256(T-25)}^{2}-0.02204(T-25)+1\left(R^{2}=0.9988\right)$$

Model validation results (Figure 2D,E) demonstrate that after thermodynamic compensation, the current-concentration response curves at different temperatures highly converge to the 25 °C baseline, effectively suppressing systematic thermal bias. In practice, the ambient temperature variable T required for Equation (4) is readily acquired from a standard, low-cost temperature sensor built into the meter’s electronic circuit board, thereby avoiding complex test strip fabrication processes.

### 3.4. Impedance-Based HCT Estimation Method

**Figure 3:**
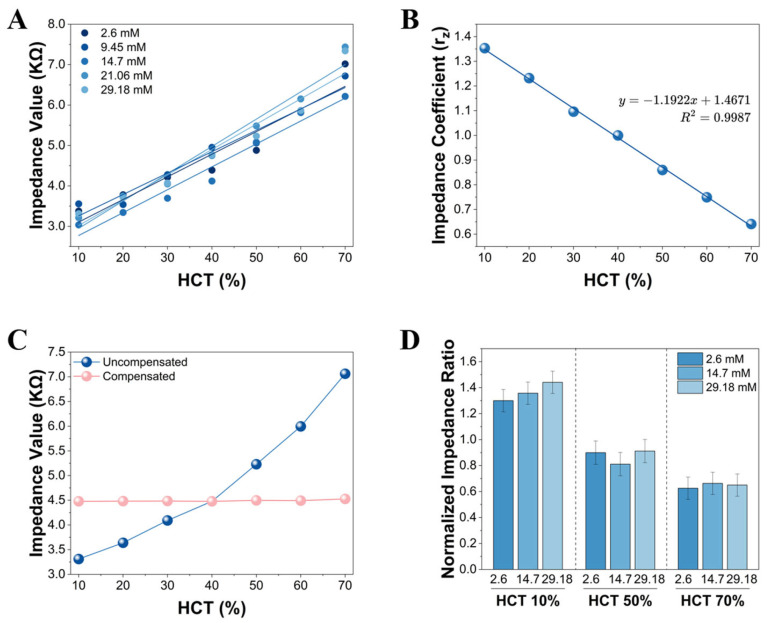
(**A**) Relationship between HCT and impedance without calibration. (**B**) Fitting relationship between the normalized impedance compensation coefficient ($r_{z}$) and HCT. Each data point represents the overall mean $r_{z}$ derived from 8 distinct glucose concentrations, with 10 independent replicates tested per concentration (total *n* = 80 per HCT level). The variable x in the equation represents HCT in decimal format). (**C**) Comparison of impedance responses at different HCT levels before and after normalization. Compared to the raw impedance (Uncompensated), the dependence of the compensated impedance (Compensated) on HCT levels is significantly reduced (*n* = 8 per HCT level). (**D**) Variations of $r_{z}$ under different glucose concentrations (using 2.6 mM, 14.7 mM, and 29.18 mM as representative examples, *n* = 10 for each test group).

In this study, a 10 kHz AC impedance signal was utilized to quantitatively estimate the HCT levels of whole blood samples. At this frequency, the RBC membrane exhibits high capacitive reactance, rendering the RBCs effective non-conductive obstacles within the current path [33,36]. Consequently, as HCT increases, the volume fraction of conductive plasma decreases and the effective current pathway becomes constricted. This results in a significant, monotonically increasing trend in the measured whole blood impedance ($Z_{meas}$), as shown in Figure 3A.

However, despite the strong correlation between impedance values and HCT, the absolute values of raw impedance exhibit minor fluctuations across different strip batches, or even among adjacent electrodes within the same batch. This is primarily attributed to batch-to-batch geometric variations inherent in the manufacturing process, such as inconsistencies in printing thickness and contact resistance. Directly utilizing raw impedance values for modeling would significantly compromise the batch-to-batch and inter-electrode robustness of the model. To address this issue, we designated 40% HCT as the standard reference state and defined the normalized impedance compensation coefficient ($r_{z}$) as the ratio of the reference impedance to the measured impedance, as presented in Equation (5):

(5) $$r_{z}=\frac{Z_{ref}}{Z_{meas}}$$

where $Z_{meas}$ represents the measured impedance of the current sample, and $Z_{ref}$ represents the average impedance corresponding to standardized 40% HCT blood for a specific batch of test strips. This ratio transformation converts the physical impedance signal into a dimensionless characteristic parameter that exclusively reflects the relative variations in HCT. In practical manufacturing, $Z_{ref}$ is established as a batch-specific constant by testing a representative sample of strips from each batch with standardized 40% HCT blood. This baseline value is then encoded into a calibration chip accompanying the strip vial, allowing the meter to automatically read and apply the batch-specific $Z_{ref}$ for normalization prior to testing.

Based on the aforementioned normalized data, the quantitative relationship between $r_{z}$ and HCT was analyzed. As shown in Figure 3B, $r_{z}$ exhibits an extremely high linear correlation with HCT across 10–70% HCT range (total *n* = 80 per HCT level). The linear regression fitting result is presented in Equation (6):

(6) $$r_{z}=-1.1922\;*\;HCT+1.4671\left(R^{2}=0.9987\right)$$

where HCT is expressed as a decimal fraction (e.g., 0.40 for 40% HCT). By solving the aforementioned fitting equation inversely, an HCT identification model was established. This model enables the system to rapidly estimate the HCT value of a sample (${HCT}_{est}$) via a single impedance measurement, providing a precise input variable for the subsequent current compensation.

To verify the accuracy of this HCT identification model, the calculated compensation coefficients were substituted back into the raw impedance data to derive the compensated impedance values (i.e., $Z_{corr}=Z_{meas}\;*\;r_{z}({HCT}_{est})$). As illustrated in Figure 3C, after model correction, the equivalent impedance values at varying HCT levels (10–70%) highly converged toward the 40% HCT baseline (mean value ≈ 4479 Ω), with significantly reduced inter-group variation. This demonstrates that the model effectively eliminates dielectric fluctuations caused by HCT variations, allowing for the precise, in situ acquisition of the HCT parameter prior to each glucose measurement.

Furthermore, in an integrated sensing system, ensuring that the HCT measurement signal is independent of the glucose detection signal is a prerequisite for achieving precise compensation. Cross-validation experiments were conducted by fixing HCT levels (e.g., 10%, 50%, and 70%) while varying glucose concentrations (ranging from a physiological low of 2.6 mM to a pathological high of 29.18 mM) and measuring the normalized impedance ratio $r_{z}$. The results (Figure 3D) indicate that at any fixed HCT level, the mean value and standard deviation of $r_{z}$ exhibited minimal variation (the coefficient of variation (CV) < 5%, across 30 independent strips from six different fabrication batches) even as the glucose concentration changed from 2.6 mM to 29.18 mM. Moreover, statistical analysis revealed that glucose concentration had no significant effect on $r_{z}$ (*p* > 0.05). These results robustly demonstrate that the 10 kHz impedance signal possesses high specificity for HCT and is decoupled from the glucose electrochemical reaction signal, thereby ensuring the reliability of the multiple correction strategy.

### 3.5. HCT Current Compensation Model and Performance Evaluation

As previously discussed, current deviations caused by HCT fluctuations are the primary cause of reduced accuracy in whole blood glucose determination. To eliminate the systematic bias induced by HCT, we established a current compensation model based on the HCT value (${HCT}_{est}$) accurately estimated in Section 3.4. The compensation strategy aims to correct the measured current ($I_{meas}$) to an equivalent current ($I_{corr}$) at the standard HCT level (${HCT}_{ref}=40\%$) by introducing an HCT compensation factor ($K_{HCT}$), as shown in Equation (7):

(7) $$I_{corr}=I_{meas}\;*\;K_{HCT}\left({HCT}_{est}\right)$$

**Figure 4:**
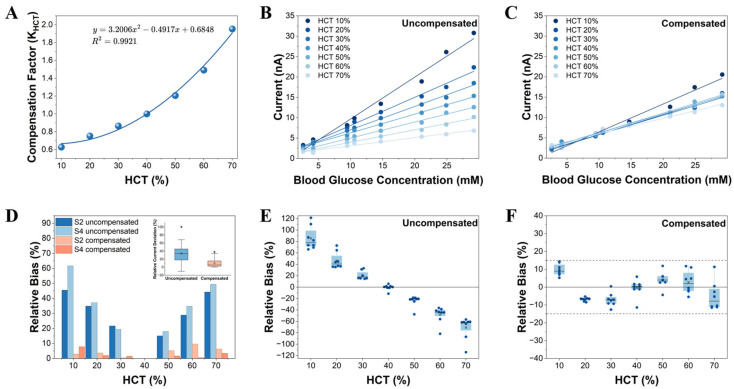
(**A**) Fitting relationship between the compensation factor ($K_{HCT}$) and the estimated HCT (${HCT}_{est}$). The independent variable x in the fitting equation represents the HCT level inputted as a scalar decimal. The goodness of fit ($R^{2}$) > 0.99 (*n* = 80 per HCT level). Current-glucose calibration curves at different HCT levels (**B**) before and (**C**) after calibration with the compensation factor. Each data point represents the mean current response derived from 10 independent replicate measurements at the corresponding HCT level and glucose concentration (*n* = 10 per point). (**D**) Relative deviation of the current response at different HCT levels before and after HCT compensation (using glucose concentrations S2 = 4.17 mM and S4 = 14.7 mM as examples). Each group conducts 10 independent tests. Inset: Distribution of the relative deviation of current response across all tested glucose concentrations and HCT conditions. After HCT compensation, the overall dispersion of the deviation distribution is significantly narrowed, indicating that the correction method effectively enhances the robustness of glucose measurement against HCT variations. (**E**,**F**) Relative bias of measured glucose concentrations compared to reference values before (**E**) and after (**F**) HCT compensation. Each scatter point represents the mean of 10 independent replicates at a specific glucose concentration. Box plots display the bias distribution across 8 distinct glucose levels per HCT group (total *n* = 80 tests per HCT level).

Through a joint fitting analysis of current data covering a wide HCT range (10–70%) and glucose concentration range (2.6 mM–29.18 mM), we adopted a quadratic polynomial function to describe the relationship between the compensation factor $K_{HCT}$ and ${HCT}_{est}$ (Figure 4A). The selection of this model balances computational complexity with fitting precision, achieving a goodness of fit ($R^{2}$) exceeding 0.99. The effectiveness of the model is further reflected in the convergence of the calibration curves. As shown in Figure 4B,C, after compensation, the originally scattered current-glucose calibration curves corresponding to different HCT levels approximate a single, highly converged baseline curve. This demonstrates that the quadratic polynomial compensation model accurately and inversely corrects the systematic interference of HCT on mass transfer kinetics.

To quantitatively evaluate the performance improvement delivered by the compensation strategy, we compared the distribution of relative bias in the current response before and after compensation. Figure 4D illustrates the deviation of the current response at different HCT levels relative to the current at the standard HCT level for specific glucose concentrations (using S2 = 4.17 mM and S4 = 14.7 mM as examples). Prior to compensation, the bias range was extensive; for samples with high HCT levels, the relative current deviation reached as high as 50%. After HCT compensation, the relative deviation of the current response narrowed significantly, proving that the accuracy and consistency of the current response across different HCT levels were fundamentally improved.

**Figure 5:**
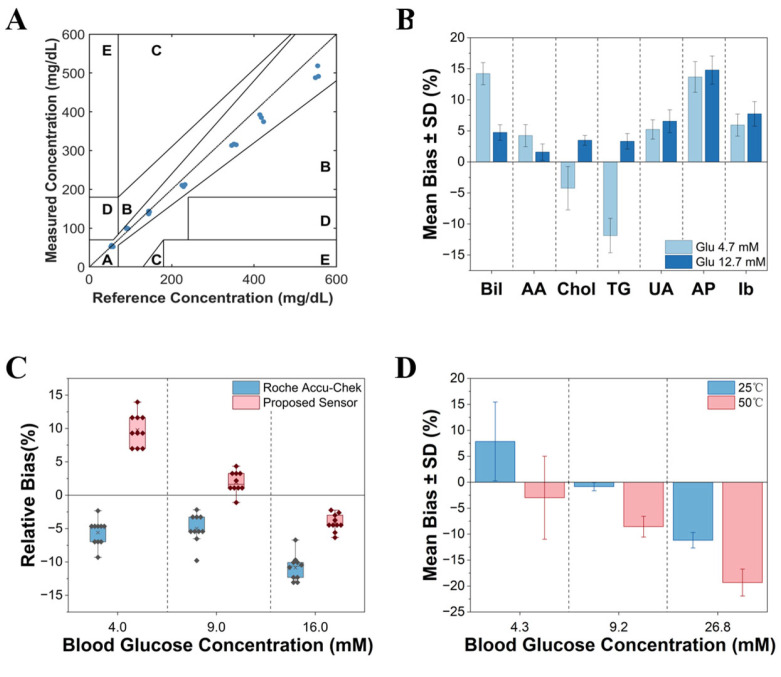
(**A**) Clarke Error Grid Analysis of the sensing system. The scatter plot displays the correlation between the glucose levels measured by the sensor and the reference values. The grid is divided into five zones based on clinical risk: Zone A: No effect on clinical action (deviation within ±20%). Zone B: Altered clinical action with little or no effect on clinical outcome. Zone C: Altered clinical action, which is likely to affect clinical outcome. Zone D: Altered clinical action, which could have significant medical risk. Zone E: Altered clinical action, which could have dangerous consequences. The clinical venous whole blood samples used in this evaluation have natural HCT levels ranging from approximately 35% to 52%. Each sample was tested utilizing a single-use test strip (*n* = 21). The results show that 100% of the data points fall within Zone A, confirming high clinical safety. (**B**) Glucose measurement interference tests for Bilirubin (Bil), Ascorbic Acid (AA), Cholesterol (Chol), Triglycerides (TG), Uric Acid (UA), Acetaminophen (AP) and Ibuprofen (Ib) at low (4.7 mM) and high (12.7 mM) glucose levels (Mean ± SD, each group conducts 5 independent tests). (**C**) Relative measurement bias of the proposed sensor versus Roche Accu-Chek in authentic clinical whole blood samples, using a biochemical analyzer as the reference (*n* = 30). (**D**) Comparison of test results between strips stored under normal conditions (25 °C, 57% RH) and those stored at 50 °C for 21 days. The bar chart represents the deviation of the measured values from the standard values (Mean ± SD, *n* = 30).

Finally, we evaluated the efficacy of the HCT compensation strategy on the final blood glucose readouts. To ensure analytical consistency, both the uncompensated and compensated current values were converted into glucose concentrations using a unified baseline calibration curve established at 40% HCT. Subsequently, the relative biases against the reference concentrations were calculated. As depicted in Figure 4E, the uncompensated measurements exhibited a pronounced HCT-dependent systematic bias: glucose levels in high-HCT samples were systematically underestimated, whereas low-HCT samples demonstrated a consistent overestimation. Following HCT compensation (Figure 4F), these deviations were substantially reduced across all tested conditions, effectively neutralizing the data dispersion induced by HCT fluctuations. The final results demonstrate that all tested samples fully comply with the error limits specified by the ISO 15197:2013 [18] standard. Clarke Error Grid analysis results (Figure 5A) showed that all data points (*n* = 21) following multiple corrections fell within the clinically acceptable Zone A (Clinically Accurate). This further confirms the reliability of the system’s measurement data from the perspective of clinical decision safety.

In summary, the strategy proposed in this study, based on impedance normalization for HCT identification and current compensation, successfully eliminated the systematic influence of HCT variations on whole blood glucose determination. The core of this strategy lies in extracting a pure dielectric response signal, which is exclusively correlated with the HCT level, from the raw impedance data. This effectively mitigates the system’s dependency on manufacturing precision, thereby enabling low-cost printed electrodes to achieve exceptional measurement accuracy and clinical applicability across a broad HCT range.

### 3.6. Validation of Analytical Performance and Stability

#### 3.6.1. Selectivity and Anti-Interference Evaluation

Endogenous or exogenous electrochemically active substances present in whole blood samples may undergo redox reactions at the electrode surface, generating false-positive current signals. To evaluate the selectivity of the sensor, we selected seven common potential interferents found in blood: Bilirubin (Bil, 90 mg/dL), Ascorbic Acid (AA, 5 mg/dL), Cholesterol (Chol, 500 mg/dL), Triglycerides (TG, 2000 mg/dL), Uric Acid (UA, 20 mg/dL), Acetaminophen (AP, 20 mg/dL) and Ibuprofen (Ib, 40 mg/dL). Interference experiments were conducted at two concentration levels: low (4.7 mM) and high (12.7 mM). As shown in Figure 5B, experimental results indicate that even in the presence of interferents at concentrations higher than normal physiological levels, the relative deviation of the sensor for glucose determination remained within 15%.

#### 3.6.2. Measurement Precision and System Accuracy

To comprehensively evaluate the analytical performance of the sensor, the intra-batch precision was first tested at low (S1: 4.3 mM), medium (S2: 9.2 mM), and high (S3: 26.8 mM) concentration levels (*n* = 10). The results showed that the CV for all samples was below 5.0%, confirming the exceptional reproducibility of the fabrication process and the multi-parameter correction algorithm. Furthermore, utilizing collected authentic clinical venous whole blood samples, we compared the measurement results of our proposed system with those of a clinically validated commercial glucometer (Roche Accu-Chek Guide Blood Glucose Meter) and a biochemical analyzer (Figure 5C). The results indicated that, compared to the biochemical analyzer, the commercial glucometer exhibited a noticeable systematic negative bias across all tested concentrations, particularly at the high concentration. In contrast, the sensor proposed in this study demonstrated a relatively smaller bias (+5% to −6%) in the medium and high concentration ranges, effectively overcoming the signal attenuation issue commonly observed in commercial products for hyperglycemic samples. All measured values across the three concentrations strictly complied with the ISO 15197:2013 [18] accuracy standard (±15%).

#### 3.6.3. Accelerated Aging Tests and Long-Term Stability

To rapidly evaluate the shelf life of the test strips, a classic accelerated aging experiment based on the Arrhenius model was conducted. The test strips were sealed and stored in a constant temperature environment at 50 °C for a 21-day thermal stress test. As shown in Figure 5D, the impact of high-temperature storage on sensor performance exhibited certain concentration-dependent characteristics:

(1) Low to Medium Concentration Range (S1 = 2.6 mM, S2 = 10.59 mM): After aging at 50 °C for 21 days, the response current of the strips to low and medium glucose concentrations showed only minor fluctuations, with measurement deviations remaining within the acceptable range. This indicates that the GDH enzyme protein maintained good structural integrity at these concentrations, and the chemical components in the dried enzyme layer remained relatively stable.
(2) High Concentration Range (S3 = 29.18 mM): At high glucose levels, the aged strips exhibited significant signal attenuation, with measured values significantly lower than the reference values (deviation > 15%). This may be attributed to high temperatures accelerating the crystallization or aggregation of the electron mediator (ferricyanide) within the microstructure, leading to increased resistance to electron transfer under high electron flux demands (i.e., high-concentration reactions); alternatively, the enzyme cofactor FAD may have partially dissociated under extreme thermal stress, reducing the maximum catalytic rate.

Nevertheless, considering that 50 °C represents an extremely harsh testing condition, the excellent performance of S1 and S2 proves that the strips have the potential for long-term stability meeting clinical requirements under conventional storage conditions. Addressing the thermal degradation in the high-concentration range, future work will focus on systematically optimizing the concentration ratios of the current protective matrix (trehalose and BSA), as well as exploring advanced polymeric encapsulation strategies (such as synthetic hydrogels) to further enhance its resistance to thermal aging.

To further evaluate the advantages of this system regarding its potential for clinical translation, we conducted a comprehensive comparison between the multiple-correction sensing system proposed in this study and other representative electrochemical glucose monitoring technologies reported in recent years (Table 1). Compared to other solutions listed in the table that rely on high-cost precious metal electrodes or additional physical filtration membranes, we achieved equivalent or superior anti-interference capabilities on inexpensive screen-printed carbon electrodes. The data indicate that this strategy covers the clinically required wide HCT range (10–70%) and broad temperature range (10–35 °C), demonstrating the technical advantages and application prospects of this system in low-cost POCT devices.

## 4. Conclusions

In summary, this study developed a high-precision, low-cost biosensing platform that addresses the long-standing challenges of HCT sensitivity and ambient temperature adaptability in electrochemical glucose monitoring. By constructing a highly integrated multi-electrode design on a single test strip and employing a time-division multiplexing dual-mode detection strategy, synergistic operation and fusion of electrochemical impedance spectroscopy and chronoamperometry were achieved within the same microfluidic channel, enabling real-time decoupling of physiological and environmental interference signals. This solution eliminates the need for expensive noble metal materials or complex post-processing, providing a concise solution for high-precision diagnosis. The core contribution of this study lies in establishing a robust algorithmic framework that utilizes normalized compensation models to eliminate the non-linear effects of temperature and HCT. Experimental results demonstrate that the system exhibits exceptional measurement stability across a wide HCT range (10–70%) and temperature range (10–35 °C). The calibrated measurements fully comply with the accuracy requirements of the ISO 15197:2013 [18] standard. Furthermore, Clarke Error Grid analysis and comparative evaluations against a commercial glucometer confirm the high clinical reliability of the proposed calibration strategy.

Furthermore, while ensuring high performance, this study fully considered the economic viability and process scalability of the product. The adoption of mature screen-printing technology and a non-precious metal electrode system greatly reduced production costs. Although there is room for optimization regarding long-term stability under extreme conditions, the excellent performance observed under conventional storage conditions has confirmed its potential for clinical application. Overall, the integrated sensing and compensation strategy proposed in this study effectively balances detection accuracy, manufacturing cost, and system complexity, providing a technological paradigm with significant translational value for the development of next-generation low-cost POCT and personal blood glucose management devices.

## Figures and Tables

**Table 1:** Comparing the performance of the sensors in this paper against other related biosensors.

Ref	Electrode Material & Mediator	Production Method	HCT Detection/Compensation Strategy	HCT Correction Range	Linear Range (mM)/LOD (μM) of Glucose
This Work	Carbon and potassium ferricyanide	Screen-printing	Impedance + Temperature Calibration	10%~70%	2.6~29.18/200
Lee et al. (2013) [37]	Carbon and potassium ferricyanide	Screen-printing	Chrono-amperometry	0%~100%	N/A (HCT Sensor Only)
Weng et al. (2017) [38]	Carbon and Ferricyanide	Screen-printing	DC pulse breakdown & Current decay ratio	9%~70%	2.2~33.7/N/A
Cinti et al. (2018) [39]	Carbon and Prussian Blue Nanoparticles	wax- and screen-printing	Physical filtration by fiber paper	N/A	0~25/170
He et al. (2019) [40]	Carbon and potassium ferricyanide	Screen-printing	Physical filtration by fiber paper	20%~70%	0~16/470
Biswas et al. (2022) [41]	N/A (Colorimetric)	Paper-based assembly	Physical filtration by glass fiber membrane	20%~65%	2.78~22.22/~2220
Cai et al. (2024)[42]	N/A (Colorimetric)	Label Printer Patterning	Physical wicking distance	25%~55%	2.5~35/~222

## Data Availability

Data will be made available on request.
